# Mindfulness and psychological well-being in adolescents: the mediating role of self-compassion, emotional dysregulation and cognitive flexibility

**DOI:** 10.1186/s40479-022-00192-y

**Published:** 2022-09-05

**Authors:** Majid Yousefi Afrashteh, Fereshteh Hasani

**Affiliations:** Department of Psychology, Faculty of Humanities, University of Zanjan, Zanjan, Iran

**Keywords:** Mindfulness, Psychological well-being, Emotional dysregulation, Self-compassion, Cognitive flexibility

## Abstract

**Introduction:**

Adolescence is a critical period of growth. Mental health during adolescence is one of the most important determinants of mental health in adulthood. The aim of this study was to analyze the relationship between mindfulness and psychological well-being of adolescents considering the mediating role of self-compassion, emotional dysregulation and cognitive flexibility.

**Methods:**

The method of this research is cross-sectional. The research population was adolescents (elementary, first and second high school) in Zanjan, Iran in 2021, whose approximate number was 14,000. Data through adolescent mindfulness questionnaires (Brown, West, Loverich, and Biegel, 2011), short form of psychological well-being questionnaire (Ryff and Keyes, 1995), short form of self-compassion scale (Raes et al., 2011), difficulty in Emotion regulation (Gratz and Roemer, 2004) and cognitive flexibility (Dennis and Vander Wal, 2010) were collected. Data analysis was performed using Pearson correlation coefficient and path analysis with SPSS-26 and lisrel-10.2 software.

**Results:**

According the results, in addition to the fact that mindfulness is directly and positively related to psychological well-being (*p <* 0.05), it is also indirectly through self-compassion and Cognitive flexibility has a positive and significant relationship with psychological well-being and also mindfulness has an indirect, negative and significant relationship with psychological well-being through emotional dysregulation (*p <* 0.05). The results supported the goodness of model fit and confirmation of hypotheses.

**Conclusion:**

Therefore, it is recommended that practitioners provide the basis for promoting psychological well-being through mindfulness, emotional dysregulation, self-compassion and cognitive flexibility.

## Introduction

Adolescence brings with it new changes in the psychological system, including thinking and planning for the future, evaluating alternatives, introspection, reasoning, abstract thinking, new levels of autonomy and assertiveness, and generally new cognitive and sexual abilities [1, 2]. In addition, adolescence lies in the context of unfavorable social conditions, including family and peer pressures, social and cultural norms, and complex demands of society and social groups, leading to prolonged adolescence and delayed adult tasks [2]. During this period, several psychological and psychiatric problems may occur [3]. In addition, factors such as violence, poverty, stigma and deprivation affect the psychological well-being of adolescents. Consequences of failing to address the mental health and psychological well-being of adolescents extend into adulthood and impair physical and mental health [4]. Therefore, one of the important influential variables of adolescence is psychological well-being [5]. According to the definition of the World Health Organization (2004), mental health or psychological well-being in terms of positive psychology is more than the absence of mental illness and is a state in which a person realizes his abilities, copes with life stresses and helps his community [6]. Ryff and Singer (1996) suggested six dimensions to psychological well-being: 1) self-acceptance, including positive feelings about past experiences and acceptance of good and bad traits; 2) Positive relationships with others including warm, reliable and satisfying relationships with others and their empathy and guidance; 3) Autonomy including self-determination, independence, resistance to pressures and also an internal source of approval; 4) Mastery of the environment, including the selection and creation of an environment tailored to psychological needs and individual values; 5) Having a purpose and direction in life; 6) Personal growth, which indicates growth and open arms for new experiences and challenges. Ryff (2018) called this six-dimensional model the Integrated Model of Personal development (IMPD) [7, 8].

One of the variables related to psychological well-being is mindfulness; one of the psychological empowerment programs is the use of mindfulness interventions (MBPs) [9]. Mindfulness can help adolescents in this difficult stage of development by providing a path to emotional well-being [10]. Mindfulness means awareness and non-judgmental awareness of what is happening in the moment [11, 12]. We are often unaware of what is happening around us, and this is the mindlessness. In contrast, mindfulness focuses our attention on the task at hand. When we are conscious, our attention is not involved in the past or the future, and we do not judge or reject what is happening now. This type of attention creates energy, transparency and happiness, and of course it is a skill that can be developed [11] intentionally or spontaneously and effortlessly [12]. A review of the research literature indicates the relationship between mindfulness and psychological well-being [13–17]. For example, in the research of Huang et al. (2021) on adolescents it was found that there is a positive relationship between mindfulness and psychological well-being and also mindfulness has a mediating role between adverse childhood experiences and psychological well-being [14]. Shankland et al. (2020) also showed that mindfulness exercises reduce anxiety and depression and increase the sense of life satisfaction that is a component of mental health and psychological well-being [16]. In another study on undergraduate students, mindfulness, self-compassion, and savoring mediated the relationship between perceived social support and psychological well-being [15]. Li et al. (2022) showed that mindfulness played a moderating role in the relationship between cyber-ostracism and adolescents’ psychological well-being and it would decrease the negative impact of cyber-ostracism on adolescents’ psychological well-being [17].

Another concept related to mindfulness and psychological well-being is self-compassion. Neff (2003) defines self-compassion as being open and compassionate with one’s sufferings, experiencing a sense of caring and kindness toward oneself, adopting a non-judgmental attitude with an understanding of one’s inadequacies and failures, and recognizing that one’s experience is part of human experience. Self-compassion is a positive emotional self-awareness that should protect one from the negative consequences of self-judgment, isolation, and rumination. Self-compassion has three main components: self-kindness (kindness and self-understanding in failures and painful situations instead of severe self-criticism); common humanity (the perception that one’s own experiences are shared among individuals in human society, rather than perceiving them separately perceptions); Mindfulness (keeping painful thoughts and feelings in balance instead of over-recognizing and exploring them) [18]. Of course, it should be noted that the component of mindfulness in self-compassion is focused on negative emotions and is not completely synonymous with the variable of mindfulness. Compassion itself is available at all times, good and bad, and can be learned [19]. The higher the level of self-compassion and mindfulness among adolescents, the higher the level of emotional well-being [10]. Ge et al. (2019) showed that Changes in mindfulness are associated with changes in self-compassion and psychological well-being among adolescents [20]. In a study conducted by Gala (2016) among adolescents, it was reported that increased mindfulness and self-compassion in adolescents are associated with changes in emotional well-being [21] and self-compassion has a mediating role between mindfulness and psychological well-being [22]. There have also been studies on the relationship between mindfulness and self-compassion and also on the relationship between self-compassion and psychological well-being along with other variables in adolescents [23–25]. For example, Múzquiz et al. (2022) reported that Self-compassion was a strong predictor of emotional well-being, and self-compassion was a partial mediator between being a victim of bullying and negative affect [24]. Also Sünbüla & Güneri (2019) showed that mindfulness is a positive and significant predictor of adolescent self-compassion [25].

Another variable associated with mindfulness and psychological well-being is the difficulty in regulating emotion also known as emotional dysregulation. Emotion regulation refers to the processes by which people pay attention to their emotions, manage the intensity and duration of emotional arousal, and change the nature and meaning of emotional states when confronted with stressful or distressing situations [26].. There are different models for describing the emotion regulation. One of them is Gross (2002) emotion regulation process model which is based on two strategies of cognitive reappraisal (CR) and expressive suppression (ES). Cognitive reappraisal is a pre-emptive strategy (before the onset of emotions) that reduces the emotional impact of the emotion-stimulating event to a positive experience by changing the interpretation and evaluation of the emotion-provoking event. Expressive suppression, on the other hand, is a response-focused strategy (after the emergence of emotions) that follows the suppression of experienced emotions [27–29]. The other one is Gratz and Roemer (2004) difficulty in emotion regulation model (DERS) that conceptualize emotion regulation as a multidimensional construct involving the (a) awareness, understanding and acceptance of emotions; (b) ability to control impulsive behaviors and engage in goal-directed behaviors when experiencing negative emotions; (c) flexible use of non-avoidant, situationally-appropriate strategies to modulate the intensity and duration of emotional responses in order to meet individual goals and situational demands; and (d) willingness to experience negative emotions in pursuit of meaningful activities in life. Difficulties in any of these areas may increase risk for psychopathology or maladaptive behaviors [30]. Some studies support the relationship between emotion regulation and psychological well-being [29, 31] and others support the relationship between mindfulness and emotion regulation [25, 32–34]. For example, Ma and Fang (2019) state that mindfulness leads to a reduction in psychological distress by reducing expressive suppression (ES) [32]. MacDonald and Baxter (2017) also recognized the difficult mediating role of emotion regulation in the relationship between mindfulness and psychological well-being [35]. The results of neurological evidence also show that the characteristics of mindfulness are highly related to the regulation of emotions and affect the way emotions are processed [33], so that mindfulness through self-compassionate behavior reduces negative self-judgment and it becomes possible for the person to use emotion regulation strategies [34].

Another construct that predicts psychological well-being is cognitive flexibility [36]. Cognitive flexibility is the ability to set aside a strategy in favor of more efficient strategies [37]. Cognitive flexibility refers to individual awareness; Awareness of the fact that there are other options in each situation. In any case, the individual has the right to choose how he or she behaves [38]. The six key components of cognitive flexibility are cognitive faulting or cognitive diffusion (the process of gradually weakening the behavioral regulation functions and verbal effects of internal events, which suggests that thoughts are nothing more than transient flow of words, thoughts, and images),acceptance (open attitude to painful inner experiences and acceptance of them), contact with the present moment (observation and description without judging the present), clarification of values, self as a context (having a stable and unchanging sense of self that thoughts and feelings are sub-aspects of self and are changing) and committed action (pursuing goals based on personal values ​​and commitment to them) [36, 39]. This feature enables a person to deal appropriately with pressures, challenges and other emotional and social issues [40]. People who think flexibly use alternative justifications and positively reconstruct their frame of mind and accept challenging situations [41]. For example, in the research of Arslan and Allen (2021), it was found that the meaning of life and cognitive flexibility mediate the effect of stress caused by the Covid-19 crisis on student well-being and reduce stress and increase psychological well-being [42]. Findings from other studies support the relationship between cognitive flexibility and mindfulness [43] and also the relationship between the three components of cognitive flexibility, self-compassion and emotional well-being [44].

**Fig. 1:**
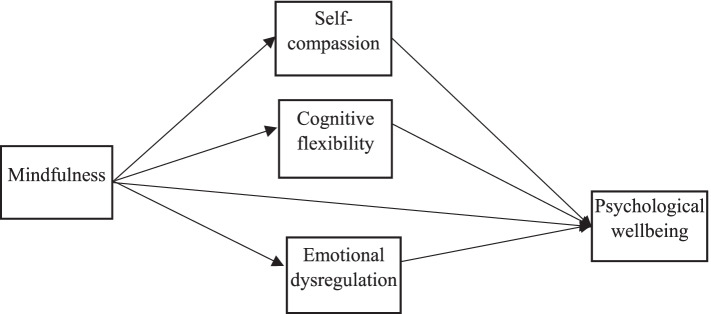
path graph for relationship between variables

Psychological well-being during adolescence is vital and one of the most important determinants of mental health in adulthood. Previous research has supported the relationship between mindfulness and psychological well-being [14, 17]. The focus of this study is to explain this relationship by studying the mediating role of three variables: self-compassion, cognitive flexibility and emotional dysregulation. Self-compassion is defined as the ability to deal with suffering and negative events without a violent and critical attitude [45]. According to Kabat-Zinn, self-compassion grows under the influence of mindfulness and can improve coping strategies and facilitate psychological well-being [45]. Similarly, emotion regulation is an inherent aspect of mindfulness literature. Emotions, especially painful ones are good goals for mindfulness that must be cultivated and accepted without judgment. The goal of mindfulness is to adapt healthier and more practical emotional processing rather than prioritizing ruminant emotions and cognitions. In addition, accepting the feeling of pain as it manifests itself facilitates relaxation therapy for coping and health [46]. Cognitive flexibility is also one of the therapeutic goals in mindfulness-based therapies such as ACT [47]. Flexibility is metaphorically as a protective shield for mental health that leads to increased mental well-being. Cognitive flexibility has been shown to have good results in improving adolescents’ psychological problems [48, 49]. The results of this study, in addition to identifying the contribution of some factors related to adolescent mental health can be useful in explaining how mindfulness is related to psychological well-being. Therefore, the main purpose of this study is to determine the mediating role of self-compassion, cognitive flexibility and emotional dysregulation in the relationship between mindfulness and psychological well-being. The conceptual model of the research is reported in Fig. 1.

## Methods

### Participants

The participants were includes adolescents aged 12 to 18 years in Zanjan, Iran 2021. The Cochran’s formula [50] was used to determine the ideal sample size, which recommended 375 participants. Since the high number of samples was emphasized in path analysis and equation model research [51], Since the high number of samples has been emphasized in path analysis and structural equation model research and considering the possibility of incomplete answers, 520 questionnaires were distributed. After discarding the questionnaires with incomplete or distorted answers, 475 questionnaires were included in the final analysis. Inclusion criteria included age range 12 to 18, no serious medical or mental illness, no use of psychiatric drugs, and no experience of grief in the past 6 months and exclusion criteria were incomplete questionnaires. The mean and standard deviation of age of participants were 15.38 and 3.78, respectively. The convenience sampling was used to select the sample. Recruitment occurred from February 27th to April 28th 2021. Details of demographic information are reported in Table 1.

**Table 1:** demographic characteristics of the subjects in the study

Variable	Frequency	Percentage
Gender		
Boy	244	51
Girl	231	49
Age		
12	42	8.8
13	60	12.6
14	61	12.8
15	75	15.7
16	67	14.1
17	86	18.1
18	84	17.6
Education level		
Elementary	47	9.89
First secondary	202	42.52
Senior secondary	226	47.57
Mother’s education		
less than a diploma	82	17.26
diploma	150	31.57
university	243	51.15
Father’s education		
less than a diploma	90	18.94
diploma	139	29.26
university	246	51.78
Number of family members		
1	174	36.63
2	167	35.15
3	93	19.57
4	41	8.63

### Procedure

In order to collect data, after obtaining the necessary permits to conduct research from the competent authorities, the preliminary stage of the research was conducted. At this stage, study questionnaires were set up for online implementation. The research sample was selected from 22 elementary, first and second secondary schools in Zanjan, which were approximately 14,000 students. After coordinating with the school staff, students or their parents were contacted and asked to participate in the study if they met their child’s inclusion criteria. The purpose of the study and the method of implementation were explained to adolescents and their parents if they were under 15 years of age. After obtaining informed consent, the link of the questionnaires was provided to those who met the inclusion criteria. Explanations were also provided regarding the non-registration of identity information and the confidentiality of participants’ information. Inclusion criteria were asked of the participants in the form of preliminary questions, and those who met the inclusion criteria were allowed access to the main questionnaires. The data obtained from the questionnaires were analyzed using Pearson correlation coefficient and path analysis with SPSS-26 and LISREL10.2 software. The executive process of this research has been approved by the Ethics Committee of Kermanshah University of Medical Sciences under No. IR.KUMS.REC.1400.608. Written informed consent was received from all participants and from their parents about adolescents under 16 years of age.

### Instruments

#### Demographics collection sheet

Participants were requested to provide the demographic information including age, level of education, Mother’s education, Father’s education and Number of family members.

#### Adolescent mindfulness short form questionnaire (MAAS-A)

In this study, the 14-item mindfulness scale [52] for adolescents was used. This questionnaire is scored based on a 6-point Likert scale from almost always to almost never, and higher scores indicate higher levels of mindfulness. Internal validity and retest indices were reported to be 0.82 and 0.79, respectively [52]. In an Iranian study, the internal consistency coefficient of the MAAS-A was 0.89 [53]. In this research, the total score of this measure has been analyzed. The reliability of this instrument in the present study was obtained with Cronbach’s alpha coefficient of 0.77.

#### Short form of psychological well-being questionnaire (PWB)

PWB has 84 question, 54 question and 18 question forms [54]. In the present study, the short 18-item form was used that has six subscales: independence; Mastery of the environment; Personal growth; Positive communication with others; purposefulness in life and self-acceptance. PWB is scored based on the Likert scale in a 6-point scale from strongly opposite to strongly agree, and the higher scores indicate psychological well-being. The test-retest reliability of the long version has been reported from 0.81 to 0.88 [55]. Also in another study, Cronbach’s alpha coefficient for this questionnaire was reported to be 0.81 [56]. Researchers in Iran have calculated the internal consistency of the 18-item short form using Cronbach’s alpha to be 0.71 and stated that this form is suitable and practical for measuring the psychological well-being of Iranian boys and girls [57]. In this research, the total score of this measure has been analyzed. The reliability of PWB in the present study was obtained with Cronbach’s alpha coefficient of 0.83.

#### Difficulty in emotion regulation scale questionnaire (DERS)

DERS [30] has 36 items and 6 subscales including non-acceptance of emotional responses; Difficulty performing purposeful behavior; Difficulty in controlling impulse; Lack of emotional awareness; Limited access to emotion regulation strategies; and lack of emotional transparency. The scale is scored on a 5 point Likert scale from very rarely to almost always. The higher scores indicate more difficulty in regulating emotion. In Gratz and Roemer research, Cronbach’s alpha coefficient has been reported to be 0.93 for the total score of this scale and between 0.80 and 0.89 for the subscales [30].. this scale has been validated in Iran and Cronbach’s alpha coefficient has been reported from 0.71 to 0.87. Therefore, the internal consistency and reliability of the Persian version of the difficulty in regulating emotion scale is confirmed [58]. In this research, the total score of this measure has been analyzed. The reliability of DERS in the present study was obtained with Cronbach’s alpha coefficient of 0.80.

#### Short form of self-compassion questionnaire (SCS-SF)

SCS-SF was developed and validated by Raes et al. (59) and is taken from the 26-items long form of Neff (60). SCS-SF has 12 items and three subscales of self-kindness, self-judgement, shared human experience - isolation; and mindfulness - an extreme simulation. This scale has been scored on a 5-point Likert spectrum from always to never [59] and researchers have calculated its internal reliability using Cronbach’s alpha to be 0.86 and stated that it has a complete correlation with the long form [9]. The use of short form is suitable for those researchers who need the overall score of their compassion scale, but measuring all 6 subscales of long form by short form is not recommended due to lower reliability [60]. Based on factor analysis of short form scale structure in Iran, Cronbach’s alpha coefficient for the whole scale and factors (self-esteem-self-judgment, shared human experience-isolation and mindfulness-extreme simulation) were 0.79, 0.68, 0.71, and 0.86 respectively and test-retest reliability was 0.90 [61]. In this research, the total score of this measure has been analyzed. The reliability of SCS-SF in the present study was obtained with Cronbach’s alpha coefficient of 0.74.

#### Cognitive flexibility questionnaire (CFI)

This scale has 20 items and includes three aspects: the desire to understand difficult situations as controllable situations; Ability to understand several alternative justifications for human life events and behavior; and measures the ability to create multiple alternative solutions to difficult situations (62). CFI is scored on a 7-point Likert spectrum from strongly disagree to strongly agree. The internal consistency of the whole questionnaire was reported to be 0.91 and the test-retest reliability was 0.81 [62]. In the Iranian sample, the internal consistency of CFI in the whole scale and the problem-solving factors and controllability perception were 0.893, 0.779 and 0.810, respectively, and the reliability of test-retest was 0.633 for the problem-solving factor and 0.766 for the control perception factor. The two-factor model of the cognitive flexibility Scale has an acceptable fit with the data, and the items probably have the power to measure the relevant factors [63]. In this research, the total score of this measure has been analyzed. The reliability of CFI in the present study was obtained with Cronbach’s alpha coefficient of 0.78.

### Statistical analysis

To analyze the data, descriptive statistical indices including mean and standard deviation as well as Pearson correlation coefficient and path analysis were used to test the hypotheses [59]. The analysis was conducted with SPSS v.26 (IBM), and LISREL v10.2. Due to the high sample size, the normality is less important. Despite this, the skew index for the research variables was between − 1 to + 1, which indicates that the distribution of variables is normal. Therefore, there was no limit to the use of Pearson correlation coefficient and path analysis with LISREL software. Path analysis with ordinal data was conducted using the diagonally weighted least squares method (WLSMV). We examined the hypothesized model using all data obtained by the original questionnaire and. The model fit indices were Chi square statistics, Chi square/df, Root Mean Square Error of Approximation (RMSEA), Comparative Fit Index (CFI), Tucker-Lewis Index [TLI, also known as the Non-normed fit index (NNFI)], Goodness of Fit Index (AGFI) and Adjusted Goodness of Fit Index (AGFI). The model was judged as having good fit when: RMSEA ≤ 0.05, CFI and TLI ≥ 0.95, and WRMR < 0.90 [60]. In terms of control variables considered, sex and age were initially considered in this study and then the control variables to be included in the model analysis will be determined based on the results of bivariate correlation between these variables and the dependent variable.

## Results

Descriptive information including mean and standard deviation and correlation matrix for relationship between variables are reported in Table 2.

**Table 2:** mean, standard deviation and correlation matrix

Variable	M	SD	1	2	3	4
1-Mindfulness	40.87	8.69	–			
2-Self-compassion	70.20	13.29	0.23^**^	–		
3-ED	104.69	22.80	−0.19^**^	− 013^**^	–	
4-Cognitive flexibility	73.42	10.04	0.17^**^	0.08	−0.11^*^	–
5-PW	53.08	9.12	0.42^**^	0.39^**^	−0.21^**^	0.38^**^

*ED* Emotional dysregulation, *PW* Psychological well-being

According to the data in Table 2, all Pearson correlation coefficients are significant for the relationship between variables (*p <* 0.05). For the control variables, age (*r* = 0.134, *p* < 0.001) were significantly correlated with Psychological well-being, while sex (*r* = 0.032, *p* = 0.092) was not significantly correlated with Psychological well-being. Therefore, age was included as control variables in the subsequent path modeling and mediation tests. The correlation coefficient results provide preliminary support for the hypotheses. The results of path analysis using Lisrel 10.2 software to determine the relationships between variables and investigate the direct effects are reported in Table 3.

**Table 3:** Indexes of model fit

Index	Value	Criteria value	Situation
X2	6.85	–	–
df	3	–	–
P	0.07	> 0.05	good
X2/df	2.28	< 3	good
RMSEA	0.05	< 0.05	good
GFI	0.99	> 0.90	good
AGFI	0.97	> 0.90	good
TLI	0.95	> 0.90	good
NFI	0.98	> 0.90	good
CFI	0.99	> 0.90	good

After incorporating age as control variable into the path analysis, the model fit indices reported in Table 3 indicated that the hypothesized model fit was well. According to the results of this table, all indices are in good condition.

**Fig. 2:**
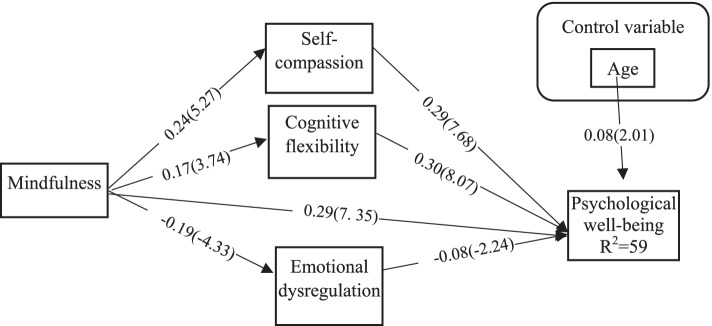
Path diagram for the relationship between variables with standard parameter and t value

Table 4 shows the direct, indirect and total effects for the relationship of the variables in the model. According to the results of this table, all direct, indirect, and total paths are significant (*P <* 0.05). Thus, self-compassion, cognitive flexibility, and difficulty in emotional dysregulation mediate the relationship between mindfulness and psychological well-being. Figure 2 shows the direct relationships.

**Table 4:** Path coefficients for direct, indirect and total effect of mindfulness, Self-compassion, Cognitive flexibility and emotional dysregulation with Psychological well-being

Paths	Standard Estimate	t-value	*P-*value
**Direct**			
Mindfulness➙PWr	0.29	7.35	*P <* 0.001
Self-compassion➙PW	0.29	7.68	*P <* 0.001
Cognitive flexibility➙PW	0.30	8.07	*P <* 0.001
ED➙PW	−0.08	− 2.24	*P =* 0.02
Mindfulness➙Self-compassion	0.24	5.27	*P <* 0.001
Mindfulness➙Cognitive flexibility	0.17	3.74	*P <* 0.001
Mindfulness➙ED	−0.19	4.33	*P <* 0.001
**Indirect**			
Mindfulness➙Self-compassion➙PW	0.069	5.33	*P <* 0.001
Mindfulness➙Cognitive flexibility➙PW	0.051	3.13	*P <* 0.01
Mindfulness➙ED➙PW	0.015	2.08	*P =* 0.03
**Total**			
Mindfulness➙PW	0.45	8.40	*P <* 0.001

*PW* Psychological well-being, *ED* Emotional dysregulation

## Discussion

The aim of this study was to determine the relationship between mindfulness and psychological well-being by analyzing the mediating role of self-compassion and emotional dysregulation and cognitive flexibility. Findings from path analysis showed that the data support hypothetical relationships between the proposed variables. The results also showed that self-compassion, emotional dysregulation and cognitive flexibility in the relationship between mindfulness and psychological well-being of adolescents have a partial mediating role. Mindfulness and psychological well-being have a direct and significant relationship.

In this study, the relationship between mindfulness and psychological well-being was confirmed. This result is consistent with those of previous research [13–17]. Huang et al. (2021) reported a positive relationship between mindfulness and psychological well-being in a group of college students [14]. Mindfulness increases happiness [64] and improves psychological well-being by increasing moment-by-moment attention without judging life situations and focusing on the present [14, 16]. Nurturing awareness and acceptance without judgment about one’s inner experiences is a rich source of self-regulation that can help people increase their psychological well-being by learning and improving self-care skills during high stress period, including adolescence [65]. For this reason, mindfulness is used as one of the psychological techniques used by many therapists; For example, adverse childhood experiences can lead to reduced psychological well-being, but mindfulness interventions can lead to improved well-being and reduce the impact of adverse childhood experiences [66].

Self-compassion mediates the relationship between mindfulness and psychological well-being so that mindfulness can indirectly lead to increased psychological well-being by increasing self-compassion. This result is consistent with the findings of Voci et al. (2018). In their research, they reported that self-compassion has a mediating role between mindfulness and the components of psychological well-being [22]. The higher the level of self-compassion and mindfulness among adolescents, the higher the level of emotional well-being [11]. Mindfulness can act as a barrier against the destructive effects of negative self-judgment and lead to more effective use of emotion regulation strategies by creating a more compassionate and kind attitude towards oneself in the face of social error or failure. Self-compassion is a substitute for self-judgment, and having a compassionate attitude toward oneself can reduce anxiety and avoidance behaviors, activate the relaxation system, and build a sense of connection, safety, and trust with others, all of which Anxiety reduction, maladaptive social interactions, and social isolation are effective [33]. In general, self-compassionate people extend the care and compassion they feel for others to others, and this improves relationships with others and other aspects of psychological well-being.

Cognitive flexibility plays a mediating role in the relationship between mindfulness and psychological well-being. Regarding the third finding, there was no study examining the mediating role of cognitive flexibility in the relationship between psychological well-being and mindfulness in an integrated model. However, in a study conducted by Wen et al. (2021) on a group of elementary school students, it was reported that cognitive flexibility mediates the relationship between mindfulness and stress and anxiety [67]. The third finding is consistent with the results of some studies on the relationship between cognitive flexibility and psychological well-being [40, 42]. In addition, it is consistent with other research findings on the relationship between mindfulness and cognitive flexibility [41]. People with high cognitive flexibility adjust their behavior more based on their values ​​and goals and less on internal events and dependencies, and also perform better by changing cognitive preferences to challenging stimuli, which in turn leads to an increase in psychological well-being of individuals.

Emotional dysregulation plays a mediating role between mindfulness and psychological well-being, meaning that mindfulness can indirectly increase psychological well-being by reducing the emotional dysregulation. This finding was consistent with the results of other studies [35, 68]. For example, MacDonald and Baxter (2017) conducted a study among a sample of college girls, concluding that the emotional dysregulation mediates the relationship between mindfulness and psychological well-being [35]. In addition, Ma et al. (2018) reported that changes of emotional dysregulation can mediate the relationship between changes in mindfulness and mental disorders [68]. Mindfulness reduces psychological distress through emotion regulation strategies [15, 69]. Adolescents at this age suffer from many stressors in their social world while trying to adapt to emotional, physical, and mental desires. Therefore, the more difficult it is to regulate emotion, the lower is the level of psychological well-being. Regulation of emotion and mindfulness can improve psychological well-being by stimulating coping mechanisms. By focusing on mindfulness, one will have a more positive attitude toward one’s abilities and will be more successful in coping with emotional consequences. Mindfulness reduces criticism and negative judgment towards the individual and leads to the acceptance of positive feelings and emotions towards the individual [65].

Regarding the clinical application of this study, it can be said that these findings can be used in various clinical situations in which adolescents are present, including schools and counseling centers. These findings can help many adolescents solve their problems and disorders. Counselors and those who interact with adolescents should do mindfulness exercises with adolescents so that adolescents can easily accept their positive and negative problems and feelings without rumination and judgment. In addition to self-compassion, the emotional dysregulation and flexibility all grows in the shadow of mindfulness, and so adolescents can accept painful feelings in their lives by promoting self-compassion and a positive view of themselves. They cultivate those who turn to them in critical situations and let go of negative thoughts. Also, with the help of flexibility, create a shield against unwanted accidents, thus reducing the damage caused by painful accidents and improving their psychological well-being.

## Conclusion

The findings of the present study confirmed the relationship between mindfulness and psychological well-being. Therefore, interventions can improve the psychological well-being and help reduce the problems of clients and also overcome the crises of adolescence. In addition, the mediating role of self-compassion, emotion regulation and cognitive flexibility was acknowledged, and it is recommended that therapists as well as those associated with adolescents consider the role of these variables. The size of the sample is large and has good external validity and generalizability. The sample of this study was relatively large and the participants were from different groups. Therefore, it can have good external validity and generalizability. Also, the age of the demographic variables after identification as a disturbing variable was entered into the path analysis to control its effect. Other demographic variables did not show a significant relationship. This supports the internal validity of the research. This study was performed on normal adolescents in Zanjan province, Iran.

There are some methodological limitations in this research that should be considered. The first limitation of this research is related to the representativeness of the sample. The results of this study were collected from schools in Zanjan. The sample selection bias may affect the generalizability of the results. It is suggested that future research be conducted in different cities of Iran to contribute to the comprehensiveness of the results. Second, the present study is of self-report, which may be affected by social desirability limitation inherent in most research [70] and there should be caution in citing the research results. Future research can minimize this problem by taking precautions to combat socially desirable responses recommended by Mick [71]. Third limitation of this research is related to its cross-sectional implementation, which limits the application of causal conclusions [72]. Researchers are advised to re-examine causal relationships through experimental or longitudinal methods. It is important to note that most studies in this area are based on cross-sectional studies and longitudinal, prospective and experimental research as well as mindfulness-based programmers over time to assess the significance of the results and the effectiveness of strategies are recommended to the researchers.

## Data Availability

The datasets during and/or analyzed during the current study available from the corresponding author on reasonable request.

## References

[1] The American Psychological Association (2002). Developing Adolescents, A Reference for Professionals.

[2] Newman BM, Newman PR (2020). Theories of Adolescent Development.

[3] Nicolson N, Ayers H (2004). Adolescent Problems.

[4] World Health Organization (WHO) (2021). Adolescent and young adult health.

[5] Piñeiro-Cossio J, Fernández-Martínez A, Nuviala A, Pérez-Ordás A. Psychological Wellbeing in Physical Education and School Sports: A Systematic Review. Enviornmental Research & Public. Health. 2021;18(3). 10.3390/ijerph18030864. 10.3390/ijerph18030864 PMC7908239 33498317

[6] World Health Organization (2004). Promoting mental health.

[7] Ryff CD, Singer B (1996). Psychological well-being: Meaning, measurement, and implications for psychotherapy research. Psychother Psychosom.

[8] Ryff CD (2018). Well-Being With Soul: Science in Pursuit of Human Potential. Perspect Psychol Sci.

[9] Galante J (2021). Mindfulness-based programmes for mental health promotion in adults in nonclinical settings: A systematic review and metaanalysis of randomised controlled trials. PLoS Med.

[10] Bluth K, Blanton PW (2014). Mindfulness and Self-Compassion: Exploring Pathways to Adolescent Emotional Well-Being. J Child Fam Stud.

[11] Germer CK (2005). Mindfulness What Is It? What Does It Matter?.

[12] Kabat-Zinn J (2005). Coming To Our Senses: Healing Ourselves and the World Through Mindfulness. New York, NY: Hyperion.

[13] Arslan U, Asici E. The mediating role of solution focused thinking in relation between mindfulness and psychological well-being in university students. Curr Psychol. 2021. 10.1007/s12144-020-01251-9.

[14] Huang C, Tan Y, Cheung SP, Hu H. Adverse Childhood Experiences and Psychological Well-Being in Chinese College Students: Mediation Effect of Mindfulness. Int J Environ Res Public Health. 2021;18(4). 10.3390/ijerph18041636. 10.3390/ijerph18041636 PMC7915366 33572110

[15] Wilson JM, Weiss A, Shook NJ. Mindfulness, self-compassion, and savoring: Factors that explain the relation between perceived social support and well-being. Personal Individ Differ. 2020;152. 10.1016/j.paid.2019.109568.

[16] Shankland R, Tessier D, Gauchet A, Baeyens C (2020). Improving Mental Health and Well-Being through Informal Mindfulness Practices: An Intervention Study. Applied Psychology: Health And Well-Being.

[17] Li X, Mu W, Wang Y, Xie P, Zhang Y, Liu T (2022). Different Roles of Rumination and Mindfulness among Cyber-Ostracized Adolescents’ Psychological Well-Being. Public Health.

[18] Neff KD (2003). Self-compassion: An alternative conceptualization of a healthy attitude toward oneself. Self Identity.

[19] Neff KD, Dahm KA, Ostafin B, Robinson M, Meier B (2015). Self-Compassion: What It Is, What It Does, and How It Relates to Mindfulness. Handbook of Mindfulness and Self-Regulation.

[20] Ge J, Wu J, Li K, Zheng Y (2019). Self-Compassion and Subjective Well-Being Mediate the Impact of Mindfulness on Balanced Time Perspective in Chinese College Students. Front Psychol.

[21] Galla BM. Within-person changes in mindfulness and self-compassion predict enhanced emotional well-being in healthy, but stressed adolescents. 2016; 49: 204-217. 10.1016/j.adolescence.2016.03.016 10.1016/j.adolescence.2016.03.016 27107398

[22] Voci A, Veneziani CHA, Fuochi G (2018). Relating Mindfulness, Heartfulness, and Psychological Well-Being: the Role of Self-Compassion and Gratitude. Mindfulness..

[23] Mendes AL, Canavarro MC, Ferreira C. The roles of self-compassion and psychological flexibility in the psychological well-being of adolescent girls. Curr Psychol. 2022. 10.1007/s12144-021-02438-4.

[24] Múzquiz J, Pérez-García AM, Bermúdez J. Relationship between Direct and Relational Bullying and Emotional Well-being among Adolescents: The role of Self-compassion. Curr Psychol. 2022. 10.1007/s12144-022-02924-3.

[25] Sünbüla ZA, Güneri OY (2019). The relationship between mindfulness and resilience: The mediating role of self compassion and emotion regulation in a sample of underprivileged Turkish adolescents. Personal Individ Differ.

[26] Thompson RA (1994). Emotion regulation: A theme in search of definition. Monogr Soc Res Child Dev.

[27] Gross JJ (2002). Emotion regulation: Affective, cognitive, and social consequences. Psychophysiology..

[28] Gross JJ (1998). Antecedent- and response-focused emotion regulation: Divergent consequences for experience, expression, and physiology. J Pers Soc Psychol.

[29] Verzeletti CH, Zammuner VL, Galli C, Agnoli S. Emotion regulation strategies and psychosocial well-being in adolescence. Cogent. Psychol. 2016;3(1). 10.1080/23311908.2016.1199294.

[30] Gratz KL, Roemer L (2004). Multidimensional Assessment of Emotion Regulation and Dysregulation: Development, Factor Structure, and Initial Validation of the Difficulties in Emotion Regulation Scale. J Psychopathol Behav Assess.

[31] Vally Z, Ahmed KH (2020). Emotion regulation strategies and psychological wellbeing: Examining cognitive reappraisal and expressive suppression in an Emirati college sample. Neurol Psychiatry Brain Res.

[32] Ma Y, Fang S. Adolescents’ Mindfulness and Psychological Distress: The Mediating Role of Emotion Regulation. Front Psychol. 2019;10. 10.3389/fpsyg.2019.01358. 10.3389/fpsyg.2019.01358 PMC6567674 31231292

[33] Deng X, Yang M, An S. Differences in frontal EEG asymmetry during emotion regulation between high and low mindfulness adolescents. Biol Psychol. 2021;158. 10.1016/j.biopsycho.2020.107990. 10.1016/j.biopsycho.2020.107990 33279594

[34] Makadi E, Koszyck D (2019). Exploring Connections Between Self-Compassion, Mindfulness, and Social Anxiety. Mindfulness..

[35] MacDonald H, Baxter E (2017). Mediators of the Relationship Between Dispositional Mindfulness and Psychological Well-Being in Female College Students. Mindfulness..

[36] Usubini AG, et al. The Impact of Psychological Flexibility on Psychological Well-Being in Adults With Obesity. Front Psychol. 2021;12. 10.3389/fpsyg.2021.636933. 10.3389/fpsyg.2021.636933 PMC8019785 33828505

[37] Scott WA (1962). Cognitive complexity and cognitive flexibility. Sociometry..

[38] Martin MM, Rubin RB. A New Measure of Cognitive Flexibility. Psychol Rep. 1995;76(2). 10.2466/pr0.1995.76.2.623.

[39] Hayes SC, Luoma JB, Bond FW, Masuda A, Lillis J (2006). Acceptance and commitment therapy: model, processes and outcomes. Behav Res Ther.

[40] Canas J, Quesada J, Antolí A, Fajardo I (2003). Cognitive flexibility and adaptability to environmental changes in dynamic complex problem-solving tasks. Ergonomics..

[41] Burton NW, Pakenham KI, Brown WJ (2010). Feasibility and effectiveness of psychosocial resilience training: a pilot study of the READY program. Psychol Health Med.

[42] Arslan G, Allen K. Exploring the association between coronavirus stress, meaning in life, psychological flexibility, and subjective well-being. Psychol Health Med. 2021. 10.1080/13548506.2021.1876892. 10.1080/13548506.2021.1876892 33487048

[43] Sinnott J, Hilton S, Wood M, Douglas D (2020). Relating Flow, Mindfulness, Cognitive Flexibility, and Postformal Thought: Two Studies. J Adult Dev.

[44] Marshall E, Brockman R (2016). The Relationships Between Psychological Flexibility, Self-Compassion, and Emotional Well-Being. J Cogn Psychother.

[45] Kabat-Zinn J (2005). Coming to our senses: Healing ourselves and the world through mindfulness.

[46] Teper R, Segal ZV, Inzlicht M (2013). Inside the mindful mind: How mindfulness enhances emotion regulation through improvements in executive control. Curr Dir Psychol Sci.

[47] Hayes SC, Levin ME, Plumb-Vilardaga J, Villatte JL, Pistorello J (2013). Acceptance and commitment therapy and contextual behavioral science: examining the progress of a distinctive model of behavioral and cognitive therapy. Behav Ther.

[48] Halliburton AE, Cooper LD (2015). Applications and adaptations of Acceptance and Commitment Therapy (ACT) for adolescents. J Contextual Behav Sci.

[49] Livheim F, Hayes L, Ghaderi A, Magnusdottir T, Högfeldt A, Rowse J, Turner S, Hayes SC, Tengström A (2015). The effectiveness of acceptance and commitment therapy for adolescent mental health: Swedish and Australian pilot outcomes. J Child Fam Stud.

[50] Cochran WG (1977). Sampling techniques.

[51] Kline RB (2005). Principles and practice of structural equation modeling.

[52] Brown KW, West AM, Loverich TM, Biegel GM (2011). Assessing adolescent mindfulness: Validation of an Adapted Mindful Attention Awareness Scale in adolescent normative and psychiatric populations. Psychol Assess.

[53] Keshmiri M, Fathi Ashtiani A, Jalali P (2019). The moderating role of mindfulness in the relationship between depression, anxiety and stress with psychological well-being. Aust J Psychol.

[54] Ryff CD, Keyes CLM (1995). The structure of psychological well-being revisited. J Pers Soc Psychol.

[55] Ryff CD (1989). Happiness is everything, or is it? Explorations on the meaning of psychological well-being. J Pers Soc Psychol.

[56] Wammerl M, Jaunig J, Mairunteregger T, Streit P (2019). The German Version of the PERMA-Profiler: Evidence for Construct and Convergent Validity of the PERMA Theory of Well-Being in German Speaking Countries. J well-being assess.

[57] Khanjani M, Shahidi S, Fathabadi J, Mazaheri MA, Shokri O (2014). Factor structure and psychometric properties of short form (18 questions) Reef psychological well-being scale in male and female students. Thought and Behavior.

[58] Besharat M (2018). Emotion adjustment difficulty scale. Thought and Behavior.

[59] Raes F, Pommier E, Neff KD, Gucht DV (2011). Construction and factorial validation of a short form of the self-compassion scale. Clin Psychol Psychother.

[60] Neff KD (2016). The Self-Compassion Scale is a Valid and Theoretically Coherent Measure of Self-Compassion. Mindfulness..

[61] Khanjani S, Foroughi AA, Sadghi K, Bahrainian SA (2016). Psychometric properties of Iranian version of self-compassion scale (short form). Pejouhandeh..

[62] Dennis JP, Vander Wal JS (2010). The Cognitive Flexibility Inventory. Instrument Development and Estimates of Reliability and Validity. Cogn Ther Res.

[63] Kohandani M, Abolmaali Alhosseini K (2017). Factor structure and psychometric properties of the Persian version of cognitive flexibility of Dennis, Vander Wal. J Psychol Methods Models.

[64] Zandi H, Amirinejhad A, Azizifar A, Aibod S, Veisani Y, Mohamadian F (2021). The effectiveness of mindfulness training on coping with stress, exam anxiety, and happiness to promote health. J Educ Health Promot.

[65] Fino E, Martoni M, Russo PM (2021). Specific mindfulness traits protect against negative effects of trait anxiety on medical student wellbeing during high-pressure periods. Adv Health Sci Educ.

[66] De Vibe M, Solhaug I, Rosenvinge JH, Tyssen R, Hanley A, Garland E. Six-year positive effects of a mindfulness-based intervention on mindfulness, coping and well-being in medical and psychology students; results from a randomized controlled trial. PLoS One. 2018;13(4). 10.1371/journal.pone.0196053. 10.1371/journal.pone.0196053 PMC5916495 29689081

[67] Wen X, Zhang Q, Liu X, Du J, Xu W. Momentary and longitudinal relationships of mindfulness to stress and anxiety among Chinese elementary school students: mediations of cognitive flexibility, self-awareness, and social environment. 2021;293:197–204. 10.1016/j.jad.2021.06.038. 10.1016/j.jad.2021.06.038 34217138

[68] Ma Y, She Z, Siu AF-Y, Zeng X, Liu X (2018). Effectiveness of Online Mindfulness-Based Interventions on Psychological Distress and the Mediating Role of Emotion Regulation. Front Psychol.

[69] Salajegheh S, Emamipoor S, Nematolah Zadeh Mahani K (2019). Structural modeling of relationship between mindfulness and psychological well-being based on mediating role of cognitive emotion regulation strategies in women with breast cancer. Quart App Psychol.

[70] Holtgraves T (2004). Social desirability and self-reports: Testing models of socially desirable responding. Personal Soc Psychol Bull.

[71] Mick DG (1996). Are studies of dark side variables confounded by socially desirable responding? The case of materialism. J Consum Res.

[72] Podsakoff NP (2003). Common method biases in behavioral research: A critical review of the literature and recommended remedies. J App Psychol.

